# Critical Analysis of the Risks in the Use of the Internet and Social Networks in Childhood and Adolescence

**DOI:** 10.3389/fpsyg.2021.683384

**Published:** 2021-07-27

**Authors:** Patricia Núñez-Gómez, Kepa Paul Larrañaga, Celia Rangel, Félix Ortega-Mohedano

**Affiliations:** ^1^Department of Applied Communication Studies, Faculty of Media and Communication Science, Complutense University of Madrid, Madrid, Spain; ^2^Complutense University of Madrid, Madrid, Spain; ^3^Department of Sociology and Communication, University of Salamanca, Salamanca, Spain

**Keywords:** childhood, adolescence, online risks, internet, social networks

## Abstract

Kids are people who consume content on the Internet very frequently and actively participate in social networks, so it is necessary to know the risks of their use by children and adolescents, in order to propose a critical analysis of them. This work is the result of two research studies: a qualitative and a quantitative analysis of 1,350 children and adolescents between 6 and 12 years old living in Spain. The objectives of this paper are analysing the attitudes of children and adolescents about the safe use of the Internet and social networks, studying the differences in the discourse of children and adults about the risks of their use, as well as defining areas for improvement to promote the safe use of the Internet and social networks. The main findings include intergenerational tensions between adults and children in the use of the Internet, the difficulty of reaching consensus and quality support when using the Internet. Children have transcended the physical and digital space considering it, so they must be given the tools, competences and security to fully develop their digital identity.

## Introduction

The health pandemic we are experiencing has changed many of our routines and consumption habits, whether due to the more or less strict lockdowns or the recommendations that we must all adopt. Children have not been an alien part of this context, but rather one more actor with unmet needs and great deficiencies (Núñez-Gómez et al., 2021). During the severe lockdowns in different countries all around the world, mobile devices have been, for many kids, their only means of socialising with their peers and entertainment, but also their learning tool to be able to follow classes (Ghungrud et al., 2021).

If the use of mobile devices has been increasing in recent years, in Spain, as a result of COVID-19, the time spent using mobile phones increased by 38% and WhatsApp by 61% (Ditrendia, 2020). These figures are in line with the academic community's growing interest in researching the uses, risks, threats and opportunities for children and, in particular, the so-called Alpha Generation (McCrindle, 2014). In this regard, we find literature from more than a decade ago by Livingstone, Ponte, Staksrud, and Núñez-Gómez (Staksrud et al., 2013; Livingstone et al., 2018; Livingstone and Blum-Ross, 2020; Núñez-Gómez et al., 2020; Livingstone and Stoilova, 2021). The studies by Kids Online, Global Kids Online, Common Sense Media, DigiLitEY Action, the work of ECREA and SIC-Spain, among others, are also a reference. In this regard, the latest study published by AIMC Niñ@s (AIMC, 2019) in Spain and carried out on children aged between 3 and 13, shows that they use an average of 4.1 devices at home, with their favourites being the smartphone, tablet, television and video console. In addition, the study shows that 39% of the children interviewed own a tablet and 27.1% own a smartphone. In terms of screen exposure, children up to 12 years old spend an average of 5 h a day in front of a screen.

According to recent studies (Núñez-Gómez et al., 2021), the Alpha Generation has a holistic experience with technology without making distinctions between formats and devices since, like adults, they do not consume in isolation. Kids under 8 years old see the Internet as a tool for entertainment and especially YouTube, which has a larger audience than many TV channels combined. In this sense, children under 12 years old love watching YouTube content produced by other peers (McRoberts et al., 2016; Yarosh et al., 2016). The use and consumption of smart screens is one of our children's favourite activities, with a relationship directly proportional to their age (Ortega-Mohedano and Pinto-Hernández, 2021).

The consumption of mobile devices is also associated with pathologies related to obesity and sedentary lifestyles (Borzekowski, 2014; Hoge et al., 2017; Kenney and Gortmaker, 2017; Robinson et al., 2017; Goodyear et al., 2018) or with other risks such as digital and physical bullying, sexting or contact with strangers (Garmendia et al., 2018; EU Kids Online, 2020). We are also facing “dangers that may be difficult to locate [such as] access to inappropriate content; uses of technology that expose the privacy and intimacy of kids to the eyes of friends and strangers” (Sádaba and Bringué, 2010, p. 88). In fact, the perception of risks differs considerably depending on the age of the children. For example, children aged 3–5 years are not aware of risks; while children aged 6–9 years have a strong desire for immediate reward, which makes them take risks (Bond and Rawlings, 2018). The EU Kids Online study (2020) highlights the following risks: excessive Internet consumption, viewing images with sexual content, sexting [receiving messages with sexual content], viewing potentially harmful user-generated content, online aggression, and cyberbullying. In channelling, detecting and solving risks, most experts agree on the decisive role of family, school and administration in making and guaranteeing a “responsible, safe, and fruitful use of technology” (Sádaba and Bringué, 2010, p. 103). Livingstone and Stoilova (2021) have recently updated the classification of online risks, taking into account whether children are related to or exposed to harmful content; whether they experience or are identified by potentially harmful contact; whether they witness/participate in and/or are victims of potentially harmful content; or whether they are party to and/or exploited by a potentially harmful contract. The mentioned classification also distinguishes between aggressive, sexual and value risks, as well as cross-cutting risks related to privacy, health and fair treatment.

Online games also have their risks. They have a negative image compared to other games (Morales, 2009) because of the addiction they generate and because they lead to diseases such as IGD (Internet Game Disorder) in some cases (Gil et al., 2020). On the other hand, online games that allow numerous players to play at the same time (Massively Multiplayer Online Role-Player Games - MMORPGs) have become very popular among young people. This type of games requires players to invest many hours, which has led to addiction problems, especially for those players who use the game as an escape from reality (Kuss et al., 2012). However, it also allows them to make friends with strangers who are playing at the same time, something that occurs mainly in boys than in girls (Bond and Rawlings, 2018). These types of games are also associated with an addiction to screens by younger children, those effects have been called “electronic cocaine” or “digital heroin” (Kardaras, 2016). Many MMORPGs are characterised by online tracking of players' sessions [referring to the process of recording, measuring and analysing people's behaviour when they browse the Internet]. The game owner can monitor when, how and with whom the game is played and, depending on the device used, the player's location, images, facial data, the use of other applications or health information can also be accessed (Corcoran and Costache, 2018). Among other things, this information will be used to make business decisions, to create consumer profiles or behavioural trends. Furthermore, Vlajic et al. (2018) argue that this type of tracking, in terms of user privacy, can be considered a major risk, because it could lead to the extraction and leakage of sensitive personal data. But it is also related to other ethical issues such as weblining [a practise that makes a user ineligible for certain goods and services based on their online profile] because, although today there is the possibility of creating anonymous avatars, with the increasing development of technology, it is very likely that, in the future, the anonymous digital avatar can be linked to the real person and their real-life transactions (Corcoran and Costache, 2018).

Privacy breaches are another risk associated with Information and Communication Technologies -ICTs (EU Kids Online, 2020). Children have difficulty understanding what privacy entails, they know little about cookies to track users and, in most cases, do not understand why personal data should not be given out, which is of particular concern to parents (Watson, 2021). In fact, it is known that children are more likely to give their personal data if they are offered a prize or reward than an adult or a teenager. In this regard, research carried out by Madden et al. (2013) suggests that adolescents are increasingly aware of what privacy entails and choose not to download certain apps when asked for personal data. On the other hand, Hernández and Ebersole (2021) highlight the different views of privacy held by children and their parents. This is compounded by the fact that children pay little, if any, attention to privacy policies and lack of understanding of the legal and economic concepts explained in them. In this regard, the UN is working to develop a General Comment on Children's Rights and the Digital Environment recognising children's rights in the digital sphere (Livingstone and Stoilova, 2021).

As for social networks, although the legal age for accessing these platforms is between 13 and 14 years old, millions of children under this age enjoy these services (Gaptain, 2020). Beyond the pandemic, in the case of Spain, the latest studies reveal that children between 9 and 16 years old consult social networks every day or very often (EU Kids Online, 2020), in primary school (Gaptain, 2020) they change their date of birth to be able to have a profile on social networks and follow influencers on preferred social networks such as TikTok, Instagram, YouTube or Twitch, while in secondary school they begin to have three types of profiles on social networks: one for the family, another one to search anonymously and freely express their opinions and the third one to spy. In this sense, according to Gaptain (2020), social networks are co-educating children through the influencers they follow, something that makes their fathers, mothers and teachers to stop being references as they become digitalized, and a turning point where the digital divide and tensions between adults and children begin to take shape.

Given that the Internet and social networks have become a place of socialisation for children and adolescents (Núñez-Gómez et al., 2020), it is necessary to propose a critical analysis that allows for the creation of a consensus on use of the Internet between children and adults. Such a consensus will only be possible in an environment of trust and mutual responsibility is built, something that does not exist today. Hence, the hypothesis of this work is that there are tensions between the preconceived ideas between adults and kids about the use of Internet and social networks by childhood and adolescence, and the demands of children and adolescents about their experience of use. Hence, the objectives of this study are as follows:

- To analyse attitudes among children and adolescents about the safe use of digital services and products.
- To study the differences between children's and adults' discourse related to risks in the use of the Internet and social networks.
- Define areas for improvement to promote safe and consensual use of the Internet and social networks.

The novelty of this work lies in responding to the need to lay the foundations for building consensus on the use of the Internet and social networks between adults and children. Children and adolescents need to be equipped with the necessary skills, competences and safety so that they can develop as responsible adults in all their facets, including the digital one. To achieve this, it is necessary to transform the current imposition of rules on the use of the Internet and social networks into commitments agreed by adults and children that promote the safe use of digital tools.

## Methodology

The complexity of analysing the reality of childhood and adolescence requires the design of research procedures that allow for an accurate approach. It is easy to fall into common places, into preconceived and idealised visions of what it is to be a child, into giving meaning to certain concepts, and into adult social representations of childhood and adolescence. Social representations establish an order in the social domain, a code, a named classification of reality, and an oriented social communication (Moscovici, 1979).

This article is presented as the result of two studies, a qualitative one to find out children's opinions on their attitudes toward the safe use of the Internet in childhood and adolescence. And another quantitative reseach, which consisted of applying a survey to children between 6 and 12 years old, to measure the use of devices and Apps in childhood and adolescence. Circumstantially, the start of the fieldwork for both studies coincided with the declaration of the pandemic due to the global health crisis caused by SARS-CoV-2. This circumstance meant that the application of the research methods selected a priori in the research design had to be adapted due to the impossibility of applying the techniques in person, and the reason for conducting the interviews (individual and group) through digital communication platforms.

For the study of social representations regarding the risks of using ICTs in childhood and adolescence, it is a requirement to apply methodologies for the identification of the elements that constitute the representations and their hierarchical organisation, as well as for the concretion and determination of the central nucleus or nuclei of these representations (Abric, 2001).

The fieldwork for the qualitative study was carried out between February and July 2020, with 16 interviews with a selected sample of experts (key informants), distributed around 6 thematic blocks: awareness, Internet hoaxes, viral challenges, influencer phenomenon, video games, and sports betting. Several field notebooks have been completed with annotations based on the observations of the adolescent co-researchers in the study. The group of co-researchers consisted of six adolescent research assistants.

The selection of key informants was based on the criteria of having a diversified sample that included different perspectives from the public, private, academic, and organised civil society spheres (Table 1). The sample included representatives from international technology companies, the Spanish State Attorney General's Office, the Guardia Civil's Telematic Crimes Group, various officials from Spanish Ministries, experts from several Spanish universities, representatives from third sector organisations, and a representative from the National Institute of Cybersecurity (INCIBE).

**Table 1:** Sample of experts by sector.

**Sectors**	**Academy**	**Public administration**	**Private company**	**NGO**	**Total**
Communication/Internet			2		2
FMCG				1	1
Security forces		1			1
Education		2	2		4
Research	3			2	5
Judicial		1	1		2
Digital leisure			1		1
**Total**	3	4	6	3	**16**

*Source: prepared by the authors*.

The adolescents participating in the group interviews belonged to several municipal participation groups of the Platform for Childhood in Spain (Table 2). They participated in six group interviews, each of which was linked to the thematic monographs addressed in the fieldwork.

**Table 2:** Sample of adolescents.

**Groups**	**Girls**	**Boys**	**Total**
Caje	2	1	3
Molina	2		2
Salamander	3		3
Gadget	3	1	4
**Total**	10	2	**12**

*Source: prepared by the authors*.

Thus, in order to analyse the risks of ICTs use by children, a qualitative study design based on Grounded Theory (Glaser and Strauss, 1967) was applied in the first phase. Above all, as it is a methodological proposal that is adjusted to the analysis of social representations for the definition of concepts as well as their properties and dimensions, and the integration of categories and subcategories into conceptual schemes. For the analysis based on Grounded Theory, Atlas.ti 9 software was used for developing the foundations of deduced, induced, and emerging categories.

Ideas and opinions in relation to the six selected thematic blocks were analysed with the collaboration of the mentioned team of adolescent co-researchers called the Gadget team. The Gadget team provided feedback during different consultation processes on the objectives of the study, the topics to be explored, the questions to ask adults and adolescents, and contributed to the interpretation of the results and the drawing of conclusions.

To consolidate the data obtained in the qualitative analysis, a second quantitative analysis was carried out through a survey on the expectations and habits of use of mobile devices and their Apps by children between 6 and 12 years old, which was conducted after the SARS- CoV-2 confinement in Spain. A self-administered questionnaire with a total of 36 questions, validated and pre-tested beforehand, was applied. At the same time, and prior to the fieldwork, an analysis of the most relevant publications of studies and research related to the object of study was carried out. The fieldwork was carried out from 23 November to 9 December 2020, with a sample of 1,350 boys and girls in the population of children aged 6–12 years in Spain. The distribution, monitoring, and follow-up of the survey was carried out through a research platform. A total of 675 surveys were administered to children between 6 and 9 years old and another 675 surveys to children betwen 10 and 12 years old. In order to be included in the study sample, the children surveyed had to be regular users of a smartphone and/or tablet, and for ethical reasons they had to have the authorisation and/or supervision of their parents and/or guardian when answering the questionnaire. Statistical processing of the data was carried out using SPSS software (version 25).

For this study, random sampling was carried out in cities with more than 10,000 inhabitants in Spain and by quotas of age, sex, region of residence, educational centre, and income received. Children from all the Autonomous Communities in Spain participated in this study.

## Results

The results of the qualitative study on attitudes toward the safe use of the Internet and social networks in childhood and adolescence are presented below, basing these results on the evidence obtained from the application of the survey on the use of devices and Apps by children.

As indicated in the chapter on methodology, a Grounded Theory analysis was carried out in order to address the type of adult representations on the type of use children make of ICTs. In order to carry out this analysis, an open coding of categories and subcategories was carried out from the interviews with adult experts, and from the ones with children and adolescents. The mentioned process resulted in the coding of 119 emerging categories in the case of the interviews with adults, and another 91 induced categories from the interviews with children and adolescents. The analysis procedure continued with the generation of an axial coding matrix that relates categories and subcategories linkable to indicated and induced phenomena from the interviews, as well as the stipulation of conditions, actions and consequences of these phenomena. In this way, the categories that provided a greater degree of explanation of the phenomena analysed according to the qualitative data from the fieldwork were verified. Furthermore, the explanatory value of these categories was double-checked by comparing the discourses of the adult informants and the adolescent informants.

From the selective coding, a number of central themes stand out that present contrasts between adult and children's discourse, as can be seen in the following Table 3.

**Table 3:** Central themes.

**Central themes**	
THEME 1	ACCESS TO INFORMATION
Central categories	Quality product design
	Critical thinking
	Internet access restriction
THEME 2	DIGITAL CITIZENSHIP
Central categories	Digital emancipation
	Opinion in childhood
	Digital socialisation
THEME 3	DIGITAL SKILLS
Central categories	Informal peer learning
	Active methodology and teacher training
	Awareness
THEME 4	CHILDREN'S SOCIAL SPACES
Central categories	Adult accompaniment
	Generating and accepting standards
	Child protagonism
THEME 5	LEGISLATION
Central categories	Legislative implementation
THEME 6	DIGITAL RIGHTS
Central categories	Entertainment/Leisure
	Exclusion from the digital environment
	Limitation of rights and freedoms
TOPIC 7	DIGITAL CULTURE
Central categories	Digital content creation
	Children's consumer rights
	Influence in social networks
THEME 8	RISKS IN THE DIGITAL ENVIRONMENT
Central categories	Addiction
	Anonymity
	Parental control
	Limitation of usage time
	Parental/peer mediation
	Generational use

*Selective coding of the analysis based on grounded theory*.

*Source: prepared by the authors*.

The main results of the quantitative analysis are detailed below, starting with children's assessment of Internet use.

**Figure 1:**
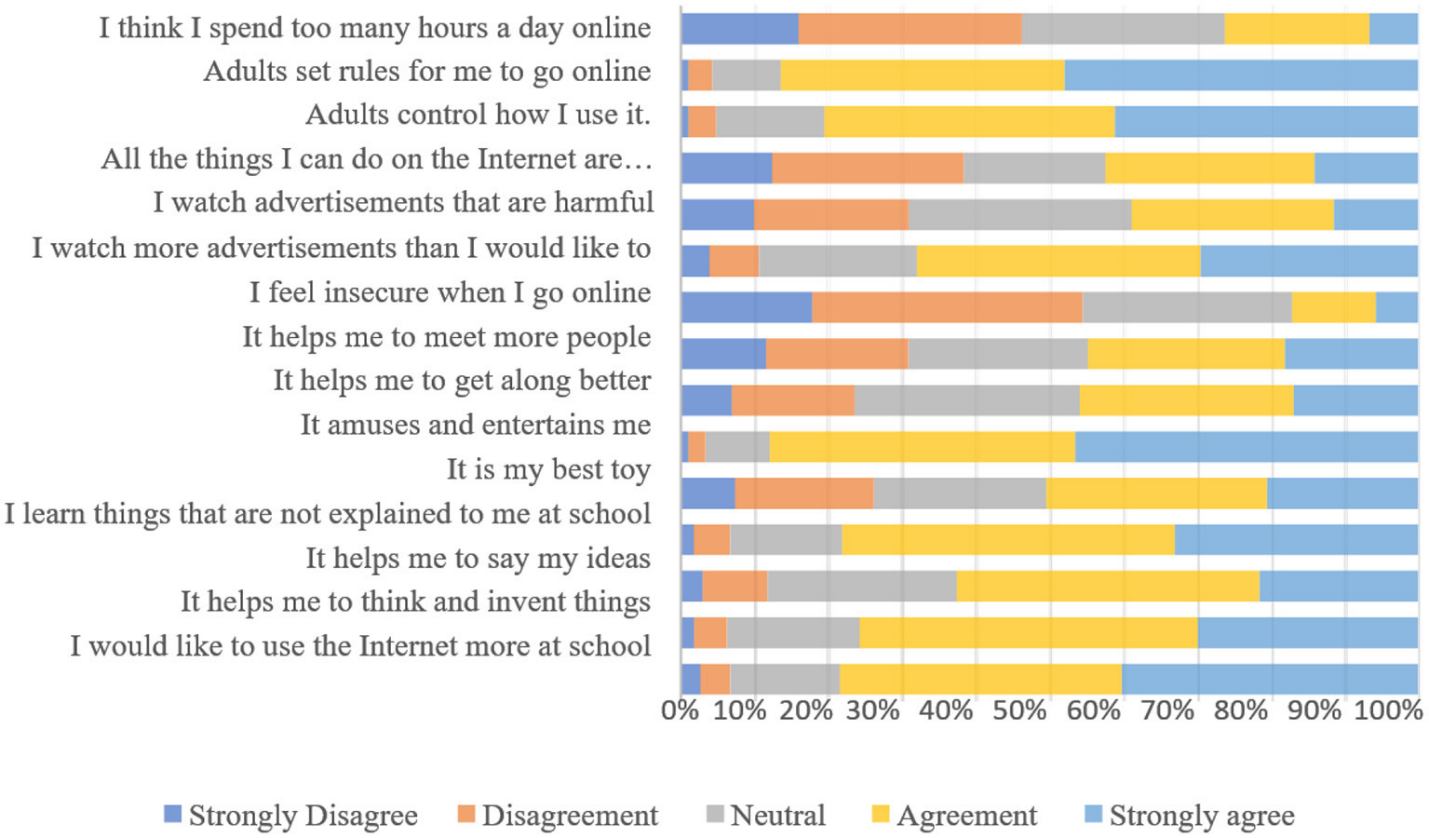
Evaluation of internet use (%). Children aged 6–12. Source: prepared by the authors.

If we look at the ratings with the highest degree of agreement in Figure 1, we can begin to foresee possible factors to be highlighted with the aggregate responses of “agree” and “strongly agree,” such as: “entertainment” with 87.9% of responses, “rules of use” and “parental control” with 86.4% and 80.5% of responses respectively, all issues linked to “education,” “learning,” specifically the use of the Internet at school and its importance for learning, with 78.4 and 78.1% of agreement on its importance, respectively. In addition, the importance given to reflection and expression of ideas and feelings is noteworthy, with 75.8 and 62.6% of the children in the sample indicating that they agree and strongly agree with these issues. Among other issues, the singular valuation of advertising on the Internet stands out, especially when assessing the amount of advertising they see. In this sense, 67.9% of children say that they see too many advertisements when they use digital products and services. In addition, 38.9% of children say that they see advertisements that they consider harmful to their development.

On the other hand, there is disagreement on other issues. Adding the responses “strongly disagree” and “disagree,” 54.5% of children say that they do not feel unsafe using the Internet, and 46.3% of children say that they do not agree that they spend many hours a day connected to the Internet.

To consolidate the analysis of children's assessment of their use of the Internet, data from a factor analysis using principal component reduction is presented. As a result of the analysis, five principal components are obtained. Table 4 shows the results obtained with the names of the principal components.

**Table 4:** Latent categories.

**Latent dimensions**	
Component 1	Socialisation
Variables:	*It helps me to speak my mind*
	*It's my best toy*
	*It helps me to relate better*
	*It helps me to get to know more people*
Component 2	Learning
Variables:	*I would like to use the Internet more at school*
	*It helps me to think and invent things*
	*It helps me to speak my mind*
	*I learn things that are not explained to me at school*
Component 3	Internet usage rules
Variables:	*Adults control me a lot about how I use it*
	*Adults set rules for me to log on*
Component 4	Quality digital consumption
Variables:	*I see more advertisements than I would like to*
	*I see advertisements that are harmful to children*
Component 5	Safety in the use of digital products/services
Variables:	*I feel insecure when I go online*
	*All the things I can do on the Internet are free of charge*

*Principal component reduction*.

*Source: prepared by the authors*.

In the procedure for extracting the latent dimensions that underpin the children's valuation of the Internet, the variable “time spent connected to the Internet” explains a very small percentage of the variance, exactly 1.99%, and is not considered in any of the principal components calculated. It is only a factor that acquires greater relevance in Component 4: Quality digital consumption, exactly in relation to the impact of advertising in the digital ecosystem and the use of digital platforms. It is necessary to pay attention to this issue as it is a key indicator in most analyses of ICTs use among children and adolescents. In any case, this issue is prioritised in order to support, along with other factors and considerations, the possible “addiction syndrome” to ICTs among children.

In the detail of the dimensions considered, we proceed to group the variables corresponding to each main component, obtaining as a result the degree of valuations given by the children in the sample to each component.

**Figure 2:**
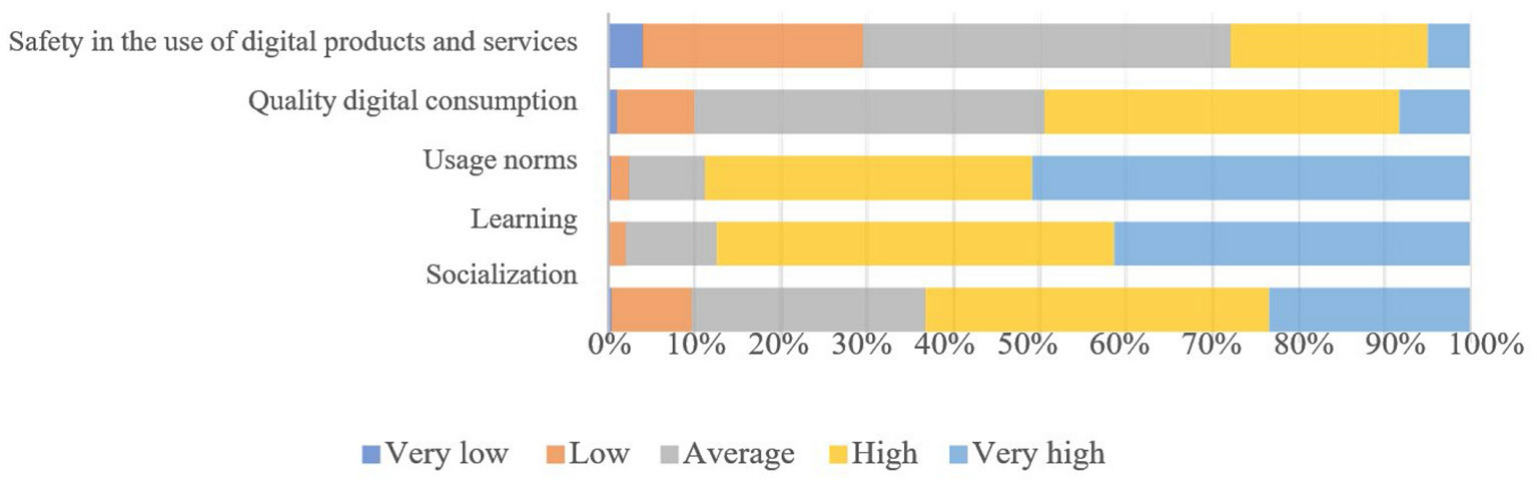
Evaluation of internet use. Main components (%). Boys and girls aged 6–12. Source: prepared by the authors.

The importance given by children to the component called “rules of use” (88.7% of children give this component a “high” and “very high” importance), “learning” (87.4% give it a “high” and “very high” rating) and “socialisation” (63.2%) stands out in Figure 2.

The number of preferred digital activities identified by the children surveyed is significant, a total of 55. Although many of these digital activities are associated with a low percentage in the preferred selection, it should be taken into account that this information was obtained through open and spontaneous responses in the questionnaire applied in the survey. The diversity of digital activities, i.e., those that can be carried out through the use of different devices, should be taken into consideration when answering the question on the time spent by children using ICTs. In this sense, the following results are relevant: (i) the different types of use that each device may have; (ii) the number of activities related to the dimensions of socialisation and learning; (iii) the role that these devices and Apps acquire for interlocution and accompaniment; (iv) and that they are devices not only to facilitate the carrying out of activities but can also be a way of being in the world and being with others in the world.

It is worth noting, as mentioned above, the importance of those digital activities linked to entertainment and social relations, and therefore included in the “socialisation” dimension, and those related to learning and access to and management of information, which are included in the “learning” dimension (Table 5).

**Table 5:** Favourite activities with devices (grouped) (%).

**Grouped activities**	**Smartphone**	**Tablet**	**Voice assistant**
Access to and management of information	4,4	3,2	30,8
Learning	3,7	9,2	6,8
Editing and audiovisual communication	5,8	4,7	0,4
Entertainment	54,9	65,6	42,3
Artistic expression	1,1	4,5	0,2
Social relations	28,6	9,2	2,1
Use of other digital products/services	1,2	0,5	7,2
NS/NC	0,4	3,0	10,2

*Boys and girls aged 6–12*.

*Source: prepared by the authors*.

Table 6 presents the relationships established in this study between the central themes obtained from the application of Grounded Theory analysis in the qualitative study and the principal components resulting from the application of a factor analysis with the data obtained from the children's survey. It has been verified which principal components were related on the basis of the categories and subcategories obtained in the Grounded Theory analysis, with the principal components.

**Table 6:** Relationships established between core themes and principal components based on grounded theory analysis.

**Central themes**	**Main components**				
	**Socialisation**	**Learning**	**Standards**	**Quality consumption**	**Security**
Access to information			X	X	X
Digital citizenship	X	X	X		
Digital skills		X	X		X
Children's social spaces	X		X		
Legislation			X		
Digital rights	X		X		X
Digital culture	X			X	
Risks in the digital environment		X	X		X

*Source: prepared by the authors*.

Based on the principal components obtained in the factor analysis of the survey data, the main results are presented below.

**Internet usage rules:**

- The adults interviewed give importance to the regulatory development in Spain and, at the same time, the deficit at the regulatory level in relation to the digital environment. On the other hand, the adolescents who took part in the research, although they did not explicitly mention legal or juridical issues, requested and demanded the recognition of their rights regarding regulations that have an impact on their daily lives. The need to reach agreements between children and adults in the generation of rules that affect their daily lives stands out. In this way, rules that are not decided and agreed between adults and children and adolescents become non-rules or imposed rules that are easy not to comply with.
- Although children are demanding their position as valid interlocutors in the processes of social interaction in all spheres and spaces, including in the cyberspace, there is still a clear opposition that corresponds to an adult imaginary in favour of limiting and restricting access to the Internet as the main strategy.
- Despite the tensions in being able to inhabit the different social spaces, the adolescent informants in the study requested and demanded the accompaniment of adults, albeit with conditions on the ways of generating and accepting the rules to be agreed upon, the latter being the main key.
- Children asked for a higher quality of adult accompaniment, and a necessary intergenerational trust between adults and children. When assessing children's attitudes in the use of ICTs, the lack of intergenerational agreements has been a generalised comment, as well as the social stigma on children due to the underestimation of their competences and aptitudes. It seems important to recognise the trust in children in order to establish partnerships between generations.

**Learning:**

- Both children and adults agree on the importance of acquiring digital skills, as ICT have a significant impact on daily lives. However, they differ in the availability and mobilisation of digital resources, in the topics of key issues affecting every day because of the use of ICTs, in the absence of a valid adult dialogue, and in the lack of real meeting places for this dialogue. Thus, the Spanish teenagers who took part in the study highlighted both the lack of digital workshops at educational centres, as well as the limited impact of changes and technological transformation in the classroom and therefore in the educational methodology.
- The main debate that is established about access to information corresponds to the degree of capacity and incompetence of children in the view of adults, giving a lower value to digital experiences and experiences acquired at any age.
- Adolescents demand quotas of responsibility, above all linked to empowerment in decision-making, and adult accompaniment, but based on agreement and respect for consensual rules.

**Socialisation:**

- The virtual environment is made up of digital social spaces where habits, values and attitudes are formed. There is a difference between digital habits and healthy habits, the former being acquired in the digital environment in relation to digital phenomena, and the latter referring to a framework and process of controlled learning of healthy digital habits.
- There is tension over the designation of territories in childhood and adolescence due to the social spaces they must inhabit. The preferred territory for children and adolescents today is cyberspace, where they develop a sense of identity and generation. In cyberspace, children strengthen their group and generational identity (for example, the importance that the movement against climate change has acquired among children and adolescents is notorious). But if for them cyberspace is the “place,” for most adults it is the “non-place” in childhood and adolescence.
- Among the adult population, there is an undervaluation of rights linked to freedom of expression or access to information, and more specifically with aspects that the adolescent informants who participated in the research consider relevant in their daily lives, such as the right to play and to enjoy their free time on the Internet.

**Quality Consumption:**

- There is a correspondence between the discourse of adults and children regarding the poor quality of digital content. The adolescent key informants in the study suggest as a necessary strategy the control of the design of digital products and therefore of the services through which they are offered.
- Digital culture is permeated by phenomena linked to the products and services of the digital ecosystem. The participating children have expressed their reticence about the control of digital companies over their data, the ways of using Apps, and the time spent using products and services that are designed to captivate the user. In addition, they point to the importance of peer pressure on the type of ICT use and intensity of use. On the other hand, for adults there are too many digital phenomena: influencers, viral challenges, online sports betting, etc., which are obvious risks for children because of their presumed low skills and incompetence.

**Safety in the use of digital products and services:**

- Adults interviewed in the study highlighted the need to limit certain civil rights of children justified by the need to prioritise other fundamental rights, essentially those linked to social and child protection rights.
- There are different types of risk and as such they are analysed and studied, to which are added gradients of greater or lesser probability of being able to suffer threats due to conditions and factors of a social and individual nature. When assessing issues related to risks on the Internet and social networks, adults consider necessary to treat children as subjects of special vulnerability. The children participating in the study are aware of the vulnerability of children and adolescents in the use of ICTs, but they also extended those risks to the rest of the generations, especially due to the conditions of use of digital products and services specifically designed to captivate the user, and the vulnerability of any user due to a key factor: the low level of digital skills of most of the population in a rapidly changing digital ecosystem. A collectivised strategy to deal with risks and threats on the Internet seems necessary.

## Discussion

Although the health pandemic has accelerated some trends that had already been noted in relation to the use of the Internet and social networks by children and adolescents, children's consumption of the Internet has doubled in recent years (EU Kids Online, 2020). Much of the content that was previously consumed on traditional channels is now consumed online, which is why YouTube is one of the platforms with the largest audience (Tur-Viñes et al., 2018). In fact, the consumption of content via the Internet and social networks is one of the favourite activities of children and adolescents (Ortega-Mohedano and Pinto-Hernández, 2021). In Spain, children connect to social networks every day (EU Kids Online, 2020) and even have different profiles in order to navigate with different identities (Gaptain, 2020). Therefore, it can be considered that the Internet and social networks have become a medium that allows children and adolescents to socialise (Núñez-Gómez et al., 2020). In addition to socialisation and entertainment, children are co-educating (Gaptain, 2020) themselves on the Internet and social networks, which means that the intergenerational digital divide between adults and children is growing, as well as the tensions it causes.

The particularity of the current research referred is that children have been given a status and position that is expressly significant both in the consultation on concepts related to the use and consumption of digital products and services and in their co-participation in the different phases of the research. In this way, it has allowed us to identify and analyse which ideas and categories are under tension and what are the potential factors of this tension at the intergenerational level. In other words, we can confirm the hypothesis of this work given that there are indeed tensions between the preconceived ideas among the adult population about the use of the Internet in childhood and adolescence, and the demands of children and adolescents about their experience of use. Hence, in terms of the objective related to analysing attitudes in childhood and adolescence about the safe use of digital services and products, we can conclude that there are numerous intergenerational tensions between the adult population and children. Firstly, there is a very tense central core due to adult conceptions of children's incapacity in terms of judgement and understanding, as well as moral incapacity. This issue affects a fundamental right such as access to information and freedom of expression. The empowerment of children is directly related to the exercise of citizenship, especially if a higher level of emancipation is required for decision-making and the achievement of intergenerational consensus in the generation of rules on the use of devices and Apps. This tension is intensified due to the difficulty of finding meeting points in relation to interests and concerns, in many cases common, and in social spaces where conversations between both population groups could be generated.

From the objective related to studying the differences between the discourse of children and adults linked to risks in the use of the Internet and social networks, the work has shown that children and adolescents have surpassed and gone beyond the threshold of traditional spaces and territories in childhood and adolescence, such as the family and school, and are situated in territories that are less controllable by adults, generating an extended conflict. The new places and territories generated by the use of the Internet extend and magnify the discourses and clichés about childhood and adolescence, understood as social constructs based on adult representation and generational order. In this way, the struggle over whether or not cyberspace should be considered an appropriate territory for children and adolescents is not resolved through restriction or strict control, but rather by considering children as valid interlocutors and subjects of rights in order to reach agreements in the formulation of rules on the use of the Internet. Children therefore demand greater appropriation of digital social spaces and social dialogue, especially where they can share with others a sense of belonging and a common construction of a way of experiencing their identity development. It can be inferred from the above that consensus is not easily generated between adults and children on the solutions provided in the face of risks on the Internet. Nor are alliances of support reached between generations to reduce the uncertainties recognised by all in the safe use of the Internet and social networks.

The tension between legitimised social representations on the safe use of the Internet and social networks by children and adolescents, and the latent and non-legitimised representations of children, requires a new social contract. A contract in which the demands of a collective, children and adolescents, who are asking to be part of the conversation and decision-making on issues that concern them, must be positivised. This contract must be based on mutualism, on an intergenerational collectivism (between adults and children) and on a generational collectivism that is involved in providing solutions to the challenge of security in the use of ICTs and in the common benefit of all groups and therefore individuals. Although it is not necessary to demonstrate the importance of theories of social representations for studies of this nature, it is evident that there are still prominent tensions between children and adults that emerge in the discourses of both social groups. Moreover, these conceptual and interpretative tensions relate to issues that are central both at the explanatory level, based on the qualitative data of this research, and in the importance of the categories, in many cases central, to which these generational tensions are linked.

In relation to the objective of defining areas for improvement in order to promote safer use of the Internet and social networks, the greatest challenge is to build real partnerships in order to reduce the recognised uncertainties in the safe use of the Internet and social networks. Above all, correcting and gradually reducing the powers and prerogatives attributed to adults in the shaping of the generational order. Therefore, it will be necessary to assess changes for intergenerational alliance and accompaniment on:

- Generate appropriate frameworks for trustworthiness and proportionality in the relationship between adults and children.
- Involve children and adolescents in the regulatory developments of the norms that concern them.
- Overcome social stigmas about child users of social networks and the Internet and their use of ICTs.
- Overcome a very deterministic view of childhood and adolescence related to the incapacitation of children's judgement, understanding and morality.
- The need for liberation and transformation of the representations held by both social groups (adults and children) in order to facilitate accompaniment as a major central category.
- Facilitate the whole set of repressed actions that exist because they are subject to collective imaginaries that each generation has of itself and of its own representation of social reality.
- Lack of appreciation and importance of issues on which there is consensus between adults and children and adolescents. There are clear consensuses, for example, the most notorious being the concern of both social groups about the risks on the Internet, or the need for mutual support for effective intergenerational accompaniment.
- The necessary increase in trust between adults and children as a necessary and initial step to support a discussion.
- The development and strengthening of generational awareness among children and adolescents and the visibility of citizenship in childhood and adolescence.
- Normalising children's agency in digital and other social spaces.
- Strengthening of accompanying partnerships to reduce recognised uncertainties in the safe use of the Internet and social networks.
- Correct and gradually reduce the powers and prerogatives attributed to adults in the shaping of a generational order.

Although it is true that, although the limitations of this work focus on a sample centred on one country and a specific number of children, the critical analysis of the risks in the use of the Internet and social networks in childhood and adolescence that this work presents provides us with great value in terms of how our society should work to tackle these risks. Today's ever-changing digital ecosystem requires us to acquire digital skills as a society even more intensely, and not just training aimed at children. We also need a globally agreed collective strategy to address risks and threats on the Internet that values children's digital space and digital culture. In fact, the SARS-CoV-2 confinement has shown that the main use that children have made of devices and Apps has been focused on learning and entertainment, and that their parents and guardians have had to relax the rules, which shows that intergenerational consensus works and that it is necessary beyond exceptional moments such as those we are experiencing due to the health pandemic.

## Data Availability Statement

The original contributions presented in the study are included in the article/supplementary material, further inquiries can be directed to the corresponding author.

## Author Contributions

PN-G and KPL were involved in the conceptualisation of the project and acquisition of data and analysis. PN-G, KPL, CR, and FO-M were involved in the interpretation of the data. All authors were involved in drafting and revising the work for intellectual content and approved the manuscript for publication.

## Conflict of Interest

The authors declare that the research was conducted in the absence of any commercial or financial relationships that could be construed as a potential conflict of interest. The handling editor declared a shared research group with one of the authors PN-G at time of review.

## Publisher's Note

All claims expressed in this article are solely those of the authors and do not necessarily represent those of their affiliated organizations, or those of the publisher, the editors and the reviewers. Any product that may be evaluated in this article, or claim that may be made by its manufacturer, is not guaranteed or endorsed by the publisher.
